# Prevalence and mortality among children with anorectal malformation: A multi-country analysis

**DOI:** 10.1002/bdr2.2129

**Published:** 2022-11-19

**Authors:** Vijaya Kancherla, Manasvi Sundar, Tandaki Lucita, Anke Lux, Marian K Bakker, Jorieke EH Bergman, Eva Bermejo-Sánchez, Mark A. Canfield, Saeed Dastgiri, Marcia L. Feldkamp, Miriam Gatt, Boris Groisman, Paula Hurtado-Villa, Kärin Kallen, Danielle Landau, Nathalie Lelong, Jorge Lopez-Camelo, Laura Elia Martinez, Pierpaolo Mastroiacovo, Margery Morgan, Osvaldo M. Mutchinick, Amy E. Nance, Wendy N. Nembhard, Anna Pierini, Antonin Sipek, Erin B. Stallings, Elena Szabova, Giovanna Tagliabue, Wladimir Wertelecki, Ignacio Zarante, Anke Rissmann

**Affiliations:** 1 Department of Epidemiology, Emory University Rollins School of Public Health, Atlanta, Georgia, USA; 2 Malformation Monitoring Centre Saxony-Anhalt, Medical Faculty, Otto-von-Guericke-University, Magdeburg, Germany; 3 Institute for Biometrics and Medical Informatics, Medical Faculty, Otto-von-Guericke-University, Magdeburg, Germany; 4 Department of Genetics, University of Groningen, University Medical Center Groningen, Eurocat Northern Netherlands, Groningen, The Netherlands; 5 ECEMC (Spanish Collaborative Study of Congenital Malformations), UIAC (Unidad de Investigación sobre Anomalías Congénitas), Instituto de Investigación de Enfermedades Raras (IIER), Instituto de Salud Carlos III, Madrid, Spain; 6 Birth Defects Epidemiology and Surveillance Branch, Texas Department of State Health Services, Austin, Texas, USA; 7 Tabriz Health Services Management Research Center, School of Medicine, Tabriz University of Medical Sciences, Tabriz, Iran; 8 Division of Medical Genetics, Department of Pediatrics, University of Utah School of Medicine, Salt Lake City, Utah, USA; 9 Malta Congenital Anomalies Registry, Directorate for Health Information and Research, Guardamangia, Malta; 10 National Network of Congenital Anomalies of Argentina (RENAC), National Center of Medical Genetics, National Administration of Laboratories and Health Institutes (ANLIS), National Ministry of Health, Buenos Aires, Argentina; 11 Department of Basic Sciences of Health, School of Health, Pontificia Universidad Javeriana Cali, Cali, Colombia; 12 National Board of Health and Welfare, Stockholm, Sweden; 13 Department of Neonatology, Soroka Medical Center, Beer-Sheva, Israel; 14 Université de Paris, Inserm U1153, Obstetrical, Perinatal and Pediatric Epidemiology Research Team (Epopé), Center for Epidemiology and Statistics Sorbonne Paris Cité (CRESS), Paris, France; 15 ECLAMC, Center for Medical Education and Clinical Research (CEMIC-CONICET), Buenos Aires, Argentina; 16 Registro DAN (Registro de Defectos al Nacimiento), Departamento de Genética, Hospital Universitario Dr. José E. González, Universidad Autónoma de Nuevo León, Monterrey, Mexico; 17 International Center on Birth Defects, International Clearinghouse for Birth Defects Surveillance and Research, Rome, Italy; 18 CARIS, the Congenital Anomaly Register for Wales, Public Health Wales, Singleton Hospital, Swansea, UK; 19 RYVEMCE, Department of Genetics, Instituto Nacional de Ciencias Médicas y Nutrición Salvador Zubirán, Mexico City, Mexico; 20 Utah Department of Health, Bureau of Children with Special Health Care Needs, Utah Birth Defects Network, Salt Lake City, Utah, USA; 21 Arkansas Center for Birth Defects Research and Prevention and Arkansas Reproductive Health Monitoring System, Fay Boozman College of Public Health, Department of Epidemiology, University of Arkansas for Medical Sciences, Little Rock, Arkansas, USA; 22 Institute of Clinical Physiology, National Research Council and Fondazione Toscana Gabriele Monasterio, Tuscany Registry of Congenital Defects, Pisa, Italy; 23 Department of Medical Genetics, Thomayer Hospital, Prague, Czech Republic; 24 Division of Birth Defects and Infant Disorders, National Center on Birth Defects and Development Disabilities, US Centers for Disease Control, Atlanta, Georgia, USA; 25 Slovak Teratologic Information Centre (FPH), Slovak Medical University, Bratislava, Slovak Republic; 26 Cancer Registry Unit, Fondazione IRCCS Istituto Nazionale dei Tumori, Lombardy, Italy; 27 OMNI-Net Ukraine Programs, Rivne, Ukraine; 28 Human Genetics Institute, Pontificia Universidad Javeriana, Bogota, Colombia and Hospital Universitario San Ignacio, Bogota, Colombia

**Keywords:** anorectal atresia, birth defect, epidemiology, mortality, prevalence

## Abstract

**Purpose:**

We examined the total prevalence, trends in prevalence, and age-specific mortality among individuals with anorectal malformation (ARM)

**Methods:**

We conducted a retrospective cohort study using data from 24 population- and hospital-based birth defects surveillance programs affiliated with the International Clearinghouse for Birth Defects Surveillance and Research (ICBDSR) from 18 countries and for births from 1974 to 2014. We estimated pooled and program-specific total prevalence per 10,000 total births. Poisson regression was used to assess time trends in prevalence from 2001 to 2012 when most programs contributed data. We calculated selected age-specific proportions of deaths, stratified by case status

**Results:**

The pooled total prevalence of ARM was 3.26 per 10,000 total births (95% Confidence Interval = 3.19, 3.32) for birth years 1974–2014. About 60% of cases were multiple or syndromic. Prevalence of multiple, syndromic, and stillborn cases decreased from 2001 to 2012. The first week mortality proportion was 12.5%, 3.2%, 28.3%, and 18.2% among all, isolated, multiple, and syndromic cases, respectively

**Conclusions:**

ARM is relatively rare, with multiple and syndromic cases showing decreasing prevalence during the study period. Mortality is a concern during the first week of life, and especially among multiple and syndromic cases. Our descriptive epidemiological findings increase our understanding of geographic variation in the prevalence of ARM and can be used to plan needed clinical services. Exploring factors influencing prevalence and mortality among individuals with ARM could inform future studies.

## 1 | INTRODUCTION

Anorectal malformation (ARM) is a rare birth defect of the gastrointestinal tract characterized by complete or partial absence of the anorectal canal with or without a perineal fistula at birth (Herman & Teitelbaum, 2012). The defect forms due to impaired development of the urogenital septum preventing the distal rectal pouch from reaching the perineum, and development of abnormal perianal muscular complex involving internal and external sphincters, and puborectalis muscle (Kluth, 2010; Ochoa, Chiesa, Vildoza, Wong, & Sepulveda, 2012). About 70% of all ARM cases present as nonsyndromic multiple congenital anomalies or as a part of a syndrome associated with other genitourinary, central nervous system, and musculoskeletal, or gastrointestinal tract defects (Solomon, 2011; Stoll, Dott, Alembik, & Roth, 2018; Totonelli et al., 2015; van de Putte et al., 2020). ARM is also seen in VATER association (vertebral anomalies, anal atresia, trachea-esophageal fistula with esophageal atresia and renal dysplasia) and VACTERL association (VATER plus cardiac and limb anomalies) (van de Putte et al., 2020). Prenatal diagnosis is both difficult and uncommon; most cases are diagnosed postnatally within 24 h of birth (Brantberg, Blaas, Haugen, Isaksen, & Eik-Nes, 2006; Ples et al., 2020; Rohrer, Vial, Gengler, Tenisch, & Alamo, 2020; Su et al., 2019). Despite receiving early surgeries, individuals with ARM face long-term health challenges involving bowel and bladder dysfunction, sexual dysfunction, and psychosocial issues (Cairo, Gasior, Rollins, & Rothstein, 2018; Rigueros Springford, Connor, Jones, Kapetanakis, & Giuliani, 2016). Affected individuals require repeated surgeries and multidisciplinary care, which are associated with high healthcare costs (Hamid, Holland, & Martin, 2007; Kovacic et al., 2018; Peña, Grasshoff, & Levitt, 2007; Tirrell, McNamara, & Dickie, 2021).

Prevalence of ARM ranges between 2 and 6 per 10,000 births (Cuschieri, 2001; Harris, Kallen, & Robert, 1995; Morris et al., 2018; Spouge & Baird, 1986; Stoll, Alembik, Roth, & Dott, 1997; Yang et al., 1994). Few registry studies have examined temporal trends in the prevalence of ARM (Lowry, Sibbald, & Bedard, 2007; Morris et al., 2018). Mortality among cases is not well examined in large birth defects registry studies; previous knowledge on mortality and survival is mostly drawn from case cohorts with small samples (Christensen, Madsen, Hauge, & Kock, 1990; Eltayeb, 2010; Haider & Fisher, 2007; Kumar et al., 2005). To the best of our knowledge, none of the studies stratified mortality outcome by case status. The objectives of our study were to examine total prevalence, trends in prevalence, and age-specific mortality among individuals with ARM, using data from multinational population- and hospital-based birth defects programs affiliated with the International Clearinghouse for Birth Defects Surveillance and Research (ICBDSR). Results were stratified by isolated, multiple, and syndromic case status.

## 2 | MATERIAL AND METHODS

### 2.1 | Design, setting, and participants

We used data from ICBDSR (http://www.icbdsr.org/), a consortium of 42 population- and hospital-based surveillance programs tracking at least one of 36 different birth defects annually in multiple countries. ICBDSR was established in 1974 and is affiliated with the World Health Organization. ICBDSR is categorized as a voluntary, nongovernmental organization with the objective to prevent birth defects and reduce the adverse outcomes related to birth defects by forming a consortium of birth defects surveillance and research programs from different countries. Each of these programs collect data on children and fetuses affected by birth defects (that serve as numerators), and the total annual number of live births and stillbirths in their source population or source hospitals (that serve as denominators), for population- or hospital-based birth defects prevalence calculations. Details on history, size of the population covered, legislation, funding, and sources of case ascertainment are documented for each program (http://www.icbdsr.org/programme-description/). Surveillance findings are compiled each year and published on the organization’s website.

In this study, we used aggregated data from 24 ICBDSR surveillance programs operating in 18 countries. Cases of ARM that resulted in live births, stillbirths, or elective terminations of pregnancy for fetal anomalies (ETOPFA), recorded between the year each surveillance program started (1974 or later), until 2014 were included. Table 1 presents characteristics of each surveillance program that contributed to the study.

### 2.2 | Case definition

The ICBDSR has defined ARM as “a congenital malformation characterized by absence of continuity of the anorectal canal or of communication between rectum and anus, or narrowing of anal canal, with or without fistula to neighboring organs. This definition excludes mild stenosis which does not need surgical intervention, and anterior anus.” The case definition corresponds to the *International Classification of Disease* (ICD)-10 codes “Q42.0 – Q42.3” and ICD-9 BPA codes “751.21 – 751.24”. Except Iran-TROCA, which also includes large intestine atresia and stenosis in their case definition of ARM, all other programs use the same case definition for ARM as defined above. Pregnancy outcome in each case was recorded as a live birth, stillbirth, or ETOPFA. Case status was examined under three categories: isolated/multiple/syndromic. Cases that presented with no other co-occurring unrelated major birth defects were classified as “isolated”. Cases that had one or more co-occurring unrelated major anomalies were classified as “multiple.” Cases that presented as a part of a genetic disorder or recognized syndrome were classified as “syndromic.”

### 2.3 | Mortality tracking

The majority of the programs followed cases with ARM from the time of birth until their discharge from the maternity or birth hospital. The follow-up was conducted by clinicians or program staff. Program-specific methods of linkage of birth defects cases to death certificates or other healthcare databases, and the maximum follow-up period, are presented in Supplement Table 1. Most programs followed cases up to at least 27 days of life and tracked mortality during this period.

### 2.4 | Statistical analysis

#### 2.4.1 | Prevalence analysis

We estimated total prevalence of ARM as the total number of cases with ARM (summing up live births, stillbirths, and ETOPFA) divided by the total births (summing up live births and stillbirths) for each participating program for the duration they contributed data. The overall total prevalence of ARM, per 10,000 total births, was estimated by combining data from all participating programs across all years where data were available. The 95% confidence interval (CI) for prevalence was estimated using the Poisson approximation of the binomial distribution. We calculated the proportion of live births, stillbirths, and ETOPFA cases with ARM, and their 95% CI, using the Poisson approximation of the binomial distribution.

#### 2.4.2 | Prevalence trend analysis

Trends in annual prevalence of ARM were examined by total, live births, stillbirths, ETOPFA, and by isolated, multiple, and syndromic case status. Random variation in prevalence trends was smoothed by combining data through overlapping sequence of three consecutive years. Poisson regression was used to quantify time trends in prevalence of ARM. Prevalence trend analysis was examined from 2001 to 2012 when most programs contributed data. Trend analyses for isolated, multiple, and syndromic ARM cases were restricted to programs that had information on case status.

#### 2.4.3 | Mortality analysis

Mortality risk was estimated as a probability measure. We examined the number of deaths among ARM cases divided by total number of live-born ARM cases. Mortality proportion was calculated by age group: Day 1/Day 2– 6/Day 7–27/Day 28–364/year 1–4/year ≥5. We also calculated mortality during the first week of life for deaths on Day 1 and Day 2–6 after birth, stratified by isolated, multiple, and syndromic case status from 18 of the 24 programs where information on case classification by co-occurring birth defects was available.

Ethics approval was provided by each surveillance program locally. Since our study had access to only the aggregated number of anorectal cases and total number of all live births in the surveillance area of each participating program, with no personal identifiers, a separate ethics approval was waived.

## 3 | RESULTS

A total of 24 programs from 18 countries contributed data for the current study. Overall period of surveillance spanned from 1974 to 2014, which varied by individual programs. Sixteen programs were population-based and eight were hospital-based. Among the 16 population-based programs, four had national, nine regional, and three had state-wide (United States) coverage. Among the eight hospital-based programs, two had national coverage and the remaining tracked births regionally (including South America-ECLAMC program which surveyed several regions in Argentina, Brazil, Uruguay, Bolivia, Chile, Ecuador, Peru, Colombia, Venezuela). Many of the programs operated in regions where ETOPFA was allowed; some programs lacked surveillance techniques to track ETOPFA cases in these regions (Table 1).

Twenty of the 24 participating programs followed the newborn until they were discharged from the maternity or birth hospital. Eleven programs were able to link to death certificates up to a specific age in some programs or up until death in others. Maximum follow-up lasted up to the first week of life in most programs; 12 programs tracked cases beyond 5 years of age (Table 2).

### 3.1 | Prevalence of ARM

A total of 9,438 cases of ARM were recorded during the study period between 1974 and 2014 in all 24 programs contributing to the study. Of these 9,438 cases, 8,583 (90.9%) were live births, 429 (4.6%) were stillbirths, and 426 (4.5%) were ETOPFA. There were 28,977,421 live births and stillbirths during the same period, serving as our total births denominator value for all contributing programs.

We estimated the pooled total prevalence of ARM during the study period (1974–2014) to be 3.26 per 10,000 total births (95% CI = 3.19, 3.32). The pooled total prevalence of ARM in 14 out of 24 programs where ETOPFA are registered was estimated to be 2.99 per 10,000 total births (95% CI = 2.91, 3.07). Iran-TROCA (9.24 per 10,000 total births), South America-ECLAMC (5.60 per 10,000 total births), and Argentina-RENAC (5.07 per 10,000 total births) had the highest estimates of total prevalence of ARM (Table 2). The total pooled prevalence of ARM during the period 2001–2012 when most programs provided data was estimated to be 3.32 per 10,000 total births (95% CI = 3.23, 3.41) (data not shown), similar to that estimated during the complete study period (1974–2014).

Over 90% of all cases were recorded as live births in programs where ETOPFA was not allowed (i.e., Latin American countries). Programs from Colombia-Bogota, Malta, and Mexico-RYVEMCE reported a relatively high proportion (>10%) of stillborn cases of ARM compared to other programs. ETOPFA was more common in some European programs, with about 30% of all ARM cases resulting in ETOPFA (Table 2).

### 3.2 | Time trends in prevalence of ARM

**FIGURE 1:**
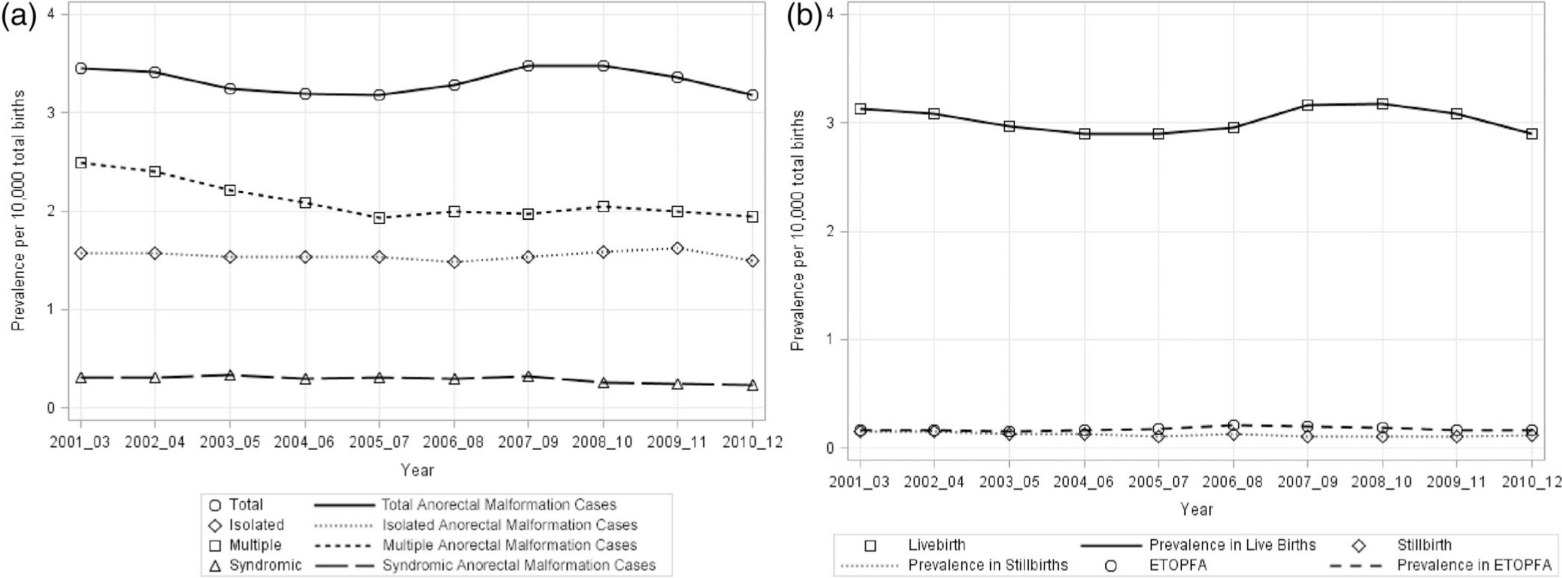
Three year rolling average prevalence trends of total, isolated, multiple, and syndromic anorectal malformation (ARM) cases (a), and by live birth, stillbirth, and elective termination of pregnancy for fetal anomalies (b) from 18 programs that contributed data to the trend analysis, International Clearinghouse for Birth Defects Surveillance and Research (ICBDSR), 2001–2012. ETOPFA = Elective Termination of Pregnancy for Fetal Anomalies.

We examined temporal trends in the prevalence of ARM for selected years (2001–2012) when most programs provided data. Eighteen programs recorded data on case status, classifying cases into isolated, multiple, and syndromic. In these 18 programs, there were no significant temporal changes in the total prevalence of all (*p* = .6859) and isolated (*p* = .8474) cases; however, total prevalence of multiple (*p* = .0004) and syndromic (*p* = .0030) cases significantly decreased among programs from 2001 to 2012. Prevalence trend graphs for total, isolated, multiple, and syndromic ARM cases from 18 programs that had data on cases status are presented in Figure 1. Additionally, for the period 2001–2012, there were no significant temporal trends for prevalence estimates restricted to live births alone (*p* = .9448) or to cases that were ETOPFA (*p* = .4908), but there was a significant decrease in the prevalence of ARM among stillborn cases (*p* = .0011) (Figure 1).

### 3.3 | Mortality in ARM

Pooled live birth prevalence of ARM from all programs during the study period (1974–2014) was 2.96 per 10,000 births, which equaled to 8,583 (91% of all cases) liveborn cases of ARM. Among programs that had data for specific follow-up times up to age 1 year, age-specific distribution of deaths showed the first week mortality proportion of 12.5%, including 654 (7.6%) that died on the day of birth, and 418 (4.9%) that died between Days 2–6 after birth (which includes Argentina for deaths occurring any time before Day 6). Further, 137 (2.0%) died between Days 7– 27, and 197 (3.1%) between Days 28–364 of age. Among programs that followed cases beyond 1 year of age, pooled data showed a lower proportion of death at 1– 4 years of age (*n* = 46; 0.9%) and ≥ 5 years of age (*n* = 27; 0.6%). There were an additional 26 deaths (0.4%) where the age at death was unknown (Table 3).

Most programs had information on death during the first week of life, and about two thirds of all deaths that occurred during the first week of life occurred on the first day of life. Argentina-RENAC, Mexico-RYVEMCE, and South America-ECLAMC reported a higher proportion of deaths in the first week of life compared to other programs (Table 3). Only 13 programs reported mortality among cases after the first year of life, and these programs were from Europe and North America. Information on mortality after the first week of life was largely unavailable for Latin American countries participating in the study. A small proportion of deaths (<1%) were reported from North American registries at ages 5 years and above.

Of the 24 programs that participated in our study, only 18 had case status information for isolated, multiple, and syndromic cases. We examined mortality by case status in these 18 programs (Table 4). First week mortality among isolated cases was 3.2%. Mortality during the first week of life was much higher among multiple (28.3%) and syndromic cases (18.2%) compared to isolated cases (Table 4).

## 4 | DISCUSSION

We conducted a large descriptive epidemiological study of ARM examining prevalence and its trends and mortality over a long span of time. Pooled data from 24 member programs of ICBDSR located in 18 countries provided a high volume of cases for this relatively rare birth defect. The total prevalence of ARM births between 1974 and 2014 was within the reported range published in previous studies, at 3.26 per 10,000 total births. Prevalence and the proportion of cases resulting in stillbirths or ETOPFA varied among programs. Total prevalence and the proportion of cases resulting in stillbirths was higher in Latin American programs. ETOPFA was more common among European programs. While we did not notice a significant change in the direction of prevalence for all cases and isolated cases, our findings showed a decrease in prevalence of multiple and syndromic cases, and stillborn cases, between 2001 and 2012. Many infants with ARM died in childhood, and among all cases, syndromic cases contributed to a higher proportion of all deaths within the first week of life.

Pooled data from EUROCAT network’s full member registries (includes only European countries) for births from 1980 to 2018 recorded the total prevalence of ARM to be 3.3 per 10,000 births (95% CI = 3.2, 3.4); the majority of these cases were live births (EUROCAT- European Surviellance of Congenital Anomalies, 2021). Another EUROCAT study, for births from 1980 to 1994, reported the total prevalence to be 3.1 per 10,000 births (Cuschieri, 2001). ICBDSR and EUROCAT networks are both large data repositories on birth defects, informed by birth defects programs in multiple countries, and some of these programs are common between the two networks (including Czech Republic, France-Paris, Germany-Saxony Anhalt, Italy-Tuscany, Malta-MCAR, Northern Netherlands, Spain-ECEMC, Sweden, Ukraine-OMNI-Net, United Kingdom-Wales). However, our analysis does not overlap completely with previous EUROCAT publications as ICBDSR also includes additional programs that are not members of the EUROCAT network and includes several programs operating outside the European Union. Specifically, our prevalence analysis presents data from multiple nonEUROCAT programs from Europe and other continents, including Argentina-RENAC, Colombia-Bogotá, Colombia-Cali, Iran-TROCA, Israel-SMC, Mexico-Nuevo Leon, Mexico-RYVEMCE, Slovak Republic, South America-ECLAMC, and USA-Arkansas, USA-Atlanta, USA-Texas and USA-Utah.

Morris et al. (2018) analyzed temporal trends in the prevalence of ARM and stenosis using data from the EUROCAT network and reported a significant increase in prevalence of cases between 2003 and 2012 (Morris et al., 2018). We did not observe an increasing trend between years 2001 and 2012 and speculate that this could be due to the differences in population characteristics and ETOPFA policies in programs contributing to EUROCAT and ICBDSR networks. Lowry et al. (2007) examined prevalence trends in ARM using data from the Alberta Congenital Anomaly Surveillance System for births occurring over a 15-year period (1990–2004) and found a nonsignificant increase in prevalence of multiple case sub-types (Lowry et al., 2007). We noted a decrease in prevalence of multiple case sub-types for the period between 2001 and 2012 in our overall pooled dataset. None of the previous studies examined prevalence trends by case status. Nevertheless, improved genetic testing may have contributed to an improved diagnosis of syndromic cases.

Few studies have examined stillbirths and mortality among infants born with ARM. Stillbirths are difficult to track in pregnancies affected by ARM as prenatal screening is not very sensitive and thus most cases are identified postnatally (Heinke et al., 2020). EUROCAT study reported a stillbirth prevalence of 0.06 per 10,000 births among ARM cases born between 2000 and 2018 (EUROCAT- European Surviellance of Congenital Anomalies, 2021). A smaller hospital-based study conducted in Norway studied 69 fetuses diagnosed prenatally with imperforate anus and documented that two of them resulted in stillbirths and 12 died after birth; babies that died had one or more additional anomalies and most of these deaths occurred either within the first 1–2 days after birth (50%) or > 2 days to 5 months of age (41%) (Brantberg et al., 2006). Nembhard, Waller, Sever, and Canfield (2001) used population-based birth defects registry data from Texas for births between 1995 and 1997 and reported the survival probability among infants with atresia or stenosis of large intestine, rectum or anus at day <1, days <7, and days <28 after birth to be 90.4%, 83.8%, and 82%, respectively (Nembhard et al., 2001). An Italian population-based study reported a 10-year survival probability of 100% for isolated cases, but survival probability was lower in nonisolated cases, especially for cases born before year 2000 (Cassina et al., 2019). Our findings agree with the results reported by some but not all of the aforementioned studies due to differences in surveillance methods and population characteristics.

It is suggested that low anomaly forms with a fistula to the perineum of ARM cases are often undiagnosed (Aldeiri et al., 2019; Amerstorfer et al., 2022; Jonker, Trzpis, & PMA, 2017). Our study may have also missed this special cases of ARM that were diagnosed after the surveillance follow-up ended, especially in programs that could not track birth defects among babies after they were discharged from the maternity hospital. Some of the differences in prevalence estimates between programs in our study may be due to this lengthy tracking limitation. Discrepancies in our findings and other study findings could also be explained by the types of follow-up employed in each of the studies. Further, it is well-documented that ARM cases undergo surgery early in life and the prognosis and life expectancy depends on early treatment, clinical complexity, and presence of multiple malformations (Hartford, Brisighelli, Gabler, & Westgarth-Taylor, 2022; Herman & Teitelbaum, 2012; Singh & Mehra, 2022). We were unable to examine the role of early surgery on mortality outcomes in the current study as it was beyond the scope of our data. Future studies could examine the impact of early surgery on survival probabilities at different ages among individuals affected by ARM.

Information on first week mortality among infants born with ARM was available for most of the programs participating in the current study. We observed that Latin American programs had a higher first week mortality among cases compared to European and North American programs participating in the study. This difference could be explored further to understand the role of prenatal screening, ETOPFA policies, and availability and access to clinical care as they can significantly influence mortality outcomes. Causes of mortality observed among multiple or syndromic ARM cases could also be further explored.

Our study has several strengths. We examined a large number of ARM cases, including all pregnancy outcomes over a 30-year period in some programs. Total cases represented live births, stillbirths, and ETOPFA. We examined age-specific mortality. We also examined mortality by isolated, multiple, and syndromic case status. Case status for ARM and other birth defects is determined and confirmed by trained surveillance personnel, increasing case specificity. Cases were tracked through multiple data sources thus increasing both internal and external validity of our findings. Data linkages between the birth defects surveillance systems and death certificates or other administrative data sources were possible for a majority of the participating programs until at least 1 week after birth and in some programs until 5 years of age.

There are several limitations with the study. Surveillance periods varied between programs. Participating programs provided data as an aggregate number of cases, live births, stillbirths, and ETOPFA, negatively impacting our ability to explore individual characteristics for analytical epidemiological studies. The definition of stillbirth varied by program, which may have introduced an undercount of cases in programs tracking stillbirths at later gestational ages. The quality of mortality data obtained from death certificates and administrative sources may vary by program and could not be validated. Linkages between programs and vital records may not be complete, potentially resulting in an undercount of mortality. It is likely that some deaths were missed due to out-migration. It was not feasible to track mortality beyond 5 years of age in most programs.

In conclusion, there is variability in the total prevalence of ARM in the programs we examined, indicating modifiable risk factors. Early life mortality is a concern, especially among multiple and syndromic cases. Our findings contribute to the knowledge of descriptive epidemiology of ARM by isolated, multiple, and syndromic cases. This information can be helpful to clinicians and stakeholders to plan needed clinical services. Considerations for surveillance attributes of participating programs may be helpful when interpreting the findings. Utilizing individual-level data and exploring factors influencing total prevalence and early life mortality among individuals with ARM could help inform future studies.

## Supplementary Material

Supplement 1

## Figures and Tables

**TABLE 1:** Description of birth defects surveillance programs contributing to the anorectal malformation (ARM) prevalence and mortality study, International Clearinghouse for Birth Defects Surveillance and Research (ICBDSR)

Country-program	Type of program	Coverage	Ascertainment period	Stillbirth definition	ETOPFA allowed	Prenatal screening services
Argentina-RENAC	Hospital-based	National	Hospital discharge	> 500 g	No	Yes, but no official program
Colombia- Bogotá	Hospital-based	Regional	first day / hospital discharge	> 500 g	Yes, since 2006, but not registered	Yes
Colombia-Cali	Hospital-based	Regional	first day / hospital discharge	> 500 g	Yes, since 2006, but not registered	Yes
Czech Republic	Population-based	National	15 years	22 weeks or > 500 g	Yes	Yes
France-Paris	Population-based	Regional	28 day	22 weeks	Yes	Yes
Germany-Saxony Anhalt	Population-based	Regional	1 years	>500 g	Yes	Yes, since 1990
Iran-TROCA	Hospital-based	Regional	1 years	20 weeks	Yes, restrictions since 2013	Yes
Israel-SMC	Hospital-based	Regional^a^	Hospital discharge	Not included	Yes, but not registered	Yes
Italy-Lombardy	Population-based	Regional	6 years	23 weeks	Yes	Yes
Italy-Tuscany	Population-based	Regional	1 years	20 weeks	Yes	Yes
Malta-MCAR	Population-based	National	1 years	22 weeks	No	Yes, gradually introduced
Mexico-Nuevo Leon	Population-based	Regional	6 days	Not included	No	Yes, only US
Mexico-RYVEMCE	Hospital-based	Regional	3 days	20 weeks or > 500 g	No	No
Netherlands-Northern	Population-based	Regional	10 years	24 weeks	Yes	Yes, since 2007
Slovak Republic	Population-based	National	Hospital discharge	> 500 g	Yes	Yes
South America- ECLAMC	Hospital-based	Regional^b^	Hospital discharge	> 500 g	No^c^	Yes
Spain-ECEMC	Hospital-based	National^d^	3 days	24 weeks or 500 grams^e^	Yes, since 1985	Yes
Sweden	Population-based	National	Until 1986:1 month, Since 1987:1 year	Until 2006:28 weeks, Since 2007:22 weeks	Yes, since 1999	Yes, since the early 1980’s
United Kingdom-Wales	Population-based	Regional	18 years	24 weeks	Yes	Yes, since 2003
Ukraine-OMNI-Net	Population-based	Regional	1 year	Until 2005:28 weeks, Since 2006:22 weeks	Yes	Yes
USA-Arkansas	Population-based	State-wide	2 years	20 weeks	Yes, till 20 weeks	Yes
USA-Atlanta	Population-based	Regional	6 years	20 weeks	Yes^f^	Yes
USA-Texas	Population-based	State-wide	1 year	20 weeks	Yes	Yes
USA-Utah	Population-based	State-wide	2 years	20 weeks	Yes	Yes

Abbreviations: ECEMC, Registry of the Spanish Collaborative Study of Congenital Malformations; ECLAMC, Latin American Collaborative Study of Congenital Malformations; ETOPFA, Elective Termination of Pregnancy for Fetal Anomalies; MCAR, Malta Congenital Anomalies Registry; OMNI-Net, Ukraine Birth Defects Prevention Program; RENAC, National Network of Congenital Anomalies of Argentina; RYVEMCE, Mexican Registry and Epidemiological Surveillance of External Congenital Malformations; SMC=Soroka Medical Center; TROCA, Tabriz Registry of Congenital Anomalies; USA, United States of America.

a Referral area of one hospital.

b Several regions in South America (Argentina, Brazil, Uruguay, Bolivia, Chile, Ecuador, Peru, Colombia, and Venezuela).

c Except for anencephaly.

d Data from all regions in Spain in the study period, currently covering around 18% of total births in the country.

e If gestational age of death is not determined (since 1980).

f ETOPFA was ascertained from prenatal diagnostic sites beginning in 1994, prior to that they were only ascertained from hospital records.

**TABLE 2:** Total prevalence of ARM per 10,000 total births, proportion of live births, stillbirths, and ETOPFA among total cases of anal atresia (for all years available in each program) from programs contributing to the study, ICBDSR

Country-program	Type of program	Surveillance period	Total births	Total cases of ARM	Total prevalence per 10,000 births (95% CI)	Live birth % (95% CI)	Stillbirth % (95% CI)	ETOPFA % (95% CI)
Argentina-RENAC^a^	Hospital-based	2009–2014	1,023,108	519	5.07 (4.65, 5.53)	93.6 (91.2, 95.6)	6.4 (4.6, 8.8)	−
Colombia-Bogota^b^	Hospital-based	2001–2014	407,199	46	1.13 (0.83,1.51)	84.8 (71.8, 92.4)	15.2 (7.6, 28.2)	−
Colombia-Cali^b^	Hospital-based	2011–2014	27,294	2	0.73 (0.07, 2.67)	100 (34.2,100)	0 (0, 65.8)	−
Czech Republic	Population-based	1994–2014	2,147,532	801	3.73 (3.48, 4.00)	95.6 (94.0, 96.8)	1.0 (0.5, 2.0)	3.4 (2.3, 4.9)
France-Paris	Population-based	1981–2014	875,241	291	3.32 (2.95, 3.73)	62.9 (57.2, 68.2)	3.8 (2.1, 6.6)	33.3 (28.2, 38.9)
Germany-Saxony Anhalt	Population-based	1980–2014	526,289	207	3.93 (3.42, 4.51)	82.1 (76.3, 86.7)	5.8 (3.3, 9.9)	12.1 (8.3,17.2)
Iran-TROCA	Hospital-based	2005–2012	141,763	131	9.24 (7.73, 10.96)	100 (97.2,100)	−	−
Israel-SMC^c^	Hospital-based	2000–2014	200,660	53	2.64 (1.98, 3.45)	100 (93.2,100)	−	−
Italy-Lombardy	Population-based	2003–2012	133,182	58	4.35 (3.31, 5.63)	79.3 (67.2, 87.8)	6.9 (2.7,16.4)	13.8 (7.2, 24.9)
Italy-Tuscany	Population-based	1992–2014	636,562	138	2.17 (1.82, 2.56)	84.8 (77.9, 89.8)	2.9 (1.1, 7.2)	12.3 (7.8,18.8)
Malta-MCAR	Population-based	1995–2013	79,948	34	4.25 (2.95, 5.94)	79.4 (63.2, 89.7)	20.6 (10.4, 36.8)	−
Mexico-Nuevo Leon	Population-based	2011–2015	442,674	19	0.43 (0.26, 0.67)	100 (83.2,100)	0 (0, 16.8)	−
Mexico-RYVEMCE	Hospital-based	1978–2013	1,198,579	538	4.49 (4.12, 4.88)	87.5 (84.5, 90.1)	12.5 (9.9,15.5)	−
Netherlands-Northern	Population-based	1981–2014	562,462	225	4.00 (3.49, 4.56)	83.1 (77.7, 87.4)	5.3 (3.1, 9.1)	11.6 (8.0,16.4)
Slovak Republic	Population-based	2001–2014	778,177	264	3.39 (3.00, 3.83)	99.2 (97.3, 99.8)	0.8 (0.2, 2.7)	0 (0,1.4)
South America- ECLAMC^a^	Hospital-based	1995–2014	2,927,555	1,638	5.60 (5.33, 5.87)	90.3 (88.8, 91.6)	9.7 (8.4,11.2)	−
Spain-ECEMC^d^	Hospital-based	1980–2013	2,891,337	587	2.03 (1.87, 2.20)	94.4 (92.2, 96.0)	5.6 (4.0, 7.8)	−
Sweden^e^	Population-based	1974–2014	4,195,523	1,330	3.17 (3.00, 3.35)	92.3 (90.8, 93.6)	0.8 (0.4,1.4)	6.9 (5.7, 8.4)
Ukraine-OMNI-Net	Population-based	2000–2013	404,172	99	2.45 (1.99, 2.98)	88.9 (81.2, 93.7)	8.1 (4.2,15.1)	3.0 (1.0, 8.5)
United Kingdom-Wales	Population-based	1998–2014	569,341	263	4.62 (4.08, 5.21)	65.4 (59.5, 70.9)	3.0 (1.6, 5.9)	31.6 (26.2, 37.4)
USA Arkansas	Population-based	1993–2012	760,777	343	4.51 (4.04, 5.01)	95.9 (93.3, 97.6)	2.9 (1.6, 5.3)	1.2 (0.5, 3.0)
USA-Atlanta^f^	Population-based	1974–2008	1,348,237	531	3.94 (3.61, 4.29)	91.3 (88.6, 93.4)	4.3 (2.9, 6.4)	4.3 (2.9, 6.4)
USA-Texas	Population-based	1996–2012	5,980,798	1,078	1.80 (1.70, 1.91)	99.1 (98.3, 99.5)	0.7 (0.4,1.5)	0.2 (0.05, 0.7)
USA-Utah	Population-based	1999–2012	719,011	243	3.38 (2.97, 3.83)	90.9 (86.7, 94.0)	1.2 (0.4, 3.6)	7.8 (5.1, 11.9)
Total		1974–2014	28,977,421	9,438	3.26 (3.19, 3.32)			

Abbreviations: CI, Confidence interval; ECEMC, Registry of the Spanish Collaborative Study of Congenital Malformations; ECLAMC, Latin American Collaborative Study of Congenital Malformations; ETOPFA, Elective Termination of Pregnancy for Fetal Anomalies; MCAR, Malta Congenital Anomalies Registry; OMNI-Net, Ukraine Birth Defects Prevention Program; RENAC, National Network of Congenital Anomalies of Argentina; RYVEMCE, Mexican Registry and Epidemiological Surveillance of External Congenital Malformations; SMC, Soroka Medical Center; TROCA, Tabriz Registry of Congenital Anomalies; USA, United States of America.

a ETOPFA not allowed.

b ETOPFA not registered.

c Data on liveborn children with ARM from one hospital.

d Spain included information on ETOPFA from 1995 to 2014. ETOPFA not routinely registered in all the participating hospitals.

e Sweden included information on ETOPFA from 1999 to 2014.

f ETOPFA were ascertained from prenatal diagnostic sites beginning in 1994, prior to that they were only ascertained from hospital records.

**TABLE 3:** Mortality in ARM affected births from programs contributing to the study for all years available in each program, ICBDSR

Country- program	Surveillance period	Number of live births with ARM	Live birth prevalence per 10,000 births (95% CI)	Age at death^a^						
				Day 1	Day 2–6	Day 7–27	Day 28- Month 12	Year 1–4	Year 5 and above	Death time unknown
				*n* (%)	*n* (%)	*n* (%)	*n* (%)	*n* (%)	*n* (%)	*n* (%)
Argentina-RENAC	2009–2014	486	4.75 (4.34, 5.19)	145 (29.8)		−	−	−	−	−
Colombia-Bogotá	2001–2014	39	0.96 (0.68, 1.31)	1 (2.6)	−	−	−	−	−	−
Colombia-Cali	2011–2014	2	0.73 (0.07, 2.67)	−	−	−	−	−	−	−
Czech Republic	1994–2014	766	3.57 (3.32, 3.83)	12 (1.6)	15 (2.0)	13 (1.7)	30 (3.9)	2 (0.3)	7 (0.9)	−
France-Paris	1981–2014	183	2.09 (1.80, 2.42)	8 (4.4)	7 (3.8)	6 (3.3)	−	−	−	0 (0)
Germany-Saxony Anhalt	1980–2014	170	3.23 (2.76, 3.75)	3 (1.8)	5 (2.9)	2 (1.2)	7 (4.1)	2 (1.2)	0 (0)	0 (0)
Iran-TROCA	2005–2012	131	9.24 (7.73, 10.96)	0 (0)	0 (0)	−	−	−	−	−
Israel-SMC^b^	2000–2014	53	2.64 (1.98, 3.45)	0 (0)	0 (0)	2 (3.8)	2 (3.8)	−	−	−
Italy-Lombardy	2003–2012	46	3.45 (2.53, 4.61)	0 (0)	0 (0)	1 (2.2)	3 (6.5)	1 (2.2)	0 (0)	0 (0)
Italy-Tuscany	1992–2014	117	1.83 (1.52, 2.20)	2 (1.7)	3 (2.6)	0 (0)	2 (1.7)	1 (0.9)	0 (0)	0 (0)
Malta-MCAR	1995–2013	27	3.38 (2.23, 4.91)	0 (0)	0 (0)	1 (3.7)	0 (0)	0 (0)	0 (0)	−
Mexico- Nuevo Leon	2011–2015	19	0.43 (0.26, 0.67)	0 (0)	2 (10.5)	1 (5.3)				1 (5.3)
Mexico- RYVEMCE	1978–2013	471	3.93 (3.58, 4.30)	85 (18.0)	22 (4.7)	−	−	−	−	2 (0.4)
Netherlands-Northern	1981–2014	187	3.32 (2.87, 3.84)	13 (7.0)	19 (10.2)	3 (1.6)	7 (3.7)	1 (0.5)	0 (0)	0 (0)
Slovak Republic	2001–2014	262	3.37 (2.97, 3.80)	0 (0)	23 (8.8)	6 (2.3)	−	−	−	−
South America	1995–2014	1,479	5.05 (4.80, 5.32)	344 (23.3)	72 (4.9)	43 (2.9)	5 (0.3)	−	−	20 (1.4)
Spain-ECEMC	1980–2013	554	1.92 (1.76, 2.08)	55 (9.9)	9 (1.6)	−	−	−	−	−
Sweden	1974–2014	1,228	2.93 (2.77, 3.10)	31 (2.5)	42 (3.4)	22 (1.8)	28 (2.3)	15 (1.2)	7 (0.6)	0 (0)
Ukraine-OMNI-Net	2000–2013	88	2.18 (1.75, 2.68)	2 (2.3)	2 (2.3)	6 (6.8)	13 (14.8)	1 (1.1)	0 (0)	2 (2.3)
United Kingdom-Wales	1998–2014	172	3.02 (2.59, 3.51)	6 (3.5)	7 (4.1)	3 (1.7)	4 (2.3)	0 (0)	0 (0)	
USA-Arkansas	1993–2012	329	4.32 (3.87, 4.82)	15 (4.6)	11 (3.3)	4 (1.2)	10 (3.0)	3 (0.9)	3 (0.9)	0 (0)
USA-Atlanta	1974–2008	485	3.60 (3.28, 3.93)	60 (12.4)	20 (4.1)	8 (1.6)	17 (3.5)	0 (0)	3 (0.6)	1 (0.2)
USA-Texas	1996–2012	1,068	1.79 (1.68, 1.90)	3 (0.3)	7 (0.7)	13(1.2)	55 (5.1)	16 (1.5)	5 (0.5)	0 (0)
USA-Utah	1999–2012	221	3.07 (2.68, 3.51)	14 (6.3)	7 (3.2)	3 (1.4)	20 (9.0)	4 (1.8)	2 (0.9)	0(0)
TOTAL	1974–2014	8,583	2.96 (2.90, 3.03)	654 (7.6^c^)	418 (4.9^c,d^)	137 (2.0^c^)	197 (3.1^c^)	46 (0.9^c^)	27 (0.6^c^)	26 (0.4^c^)

Abbreviations: ECEMC, Registry of the Spanish Collaborative Study of Congenital Malformations; ECLAMC, Latin American Collaborative Study of Congenital Malformations; ETOPFA, Elective Termination of Pregnancy for Fetal Anomalies; MCAR, Malta Congenital Anomalies Registry; OMNI-Net, Ukraine Birth Defects Prevention Program; RENAC, National Network of Congenital Anomalies of Argentina; RYVEMCE, Mexican Registry and Epidemiological Surveillance of External Congenital Malformations; SMC, Soroka Medical Center; TROCA, Tabriz Registry of Congenital Anomalies; USA, United States of America.

a A hyphen (–) means that the registry did not report follow-up data for that time period.

b Data on livebom children with anorectal atresia from one hospital.

c Excludes programs that have no data on mortality for selected age at death.

d Frequency and percentage refer to first week mortality.

**TABLE 4:** First week mortality by case status among live births affected with ARM for all years available from 18 programs contributing to the study, ICBDSR

Country-registry	Isolated ARM^a^						Multiple ARM^a^						Syndromic ARM^a^					
	Total isolated cases	Type of birth			Mortality in LB		Total multiple cases	Type of birth			Mortality in LB		Total syndromic cases	Type of birth			Mortality in LB	
		ETOPFA	SB	LB	Day 1	Day 2–6		ETOPFA	SB	LB	Day 1	Day 2–6		ETOPFA	SB	LB	Day 1	Day 2–6
	*n* (%)	*n* (%)	*n* (%)	*n* (%)	*n* (%)	*n* (%)	*n* (%)	*n* (%)	*n* (%)	*n* (%)	*n* (%)	*n* (%)	*n* (%)	*n* (%)	*n* (%)	*n* (%)	*n* (%)	*n* (%)
Argentina-RENAC^b^	212 (40.9)	−	9 (4.2)	203 (95.8)	26 (12.8)^c^		270 (52.0)	−	23 (8.5)	247 (91.5)	115 (46.6)^c^		37 (7.1)	−	1 (2.7)	36 (97.3)	4 (11.1)^c^	
Colombia-Bogotá^d^	19 (41.3)	−	2 (10.5)	17 (89.5)	0 (0)	−	15 (32.6)	−	4 (26.7)	11 (73.3)	1 (9.1)	−	12 (26.1)	−	1 (8.3)	11 (91.7)	0 (0)	−
Colombia-Cali	2 (100)	−	0 (0)	2 (100)	−	−	0 (0)	−	0 (0)	0 (0)	−	−	0 (0)	−	0 (0)	0 (0)	−	−
France-Paris	106 (36.4)	0 (0)	1 (0.9)	105 (99.1)	2 (1.9)	0 (0)	134 (46.1)	69 (51.5)	6 (4.5)	59 (44.0)	6 (10.2)	6 (10.2)	51 (17.5)	28 ( 54.9)	4 (7.8)	19 (37.3)	0 (0)	1 (5.3)
Germany-Saxony Anhalt	100 (48.3)	2 (2.0)	3 (3.0)	95 (95.0)	0 (0)	0 (0)	93 (44.9)	19 (20.4)	8 (8.6)	66 (71.0)	2 (3.0)	4 (6.1)	14 (6.8)	4 (28.6)	1 (7.1)	9 (64.3)	1 (11.1)	1 (11.1)
Israel-SMC^e^	44 (83.0)	−	−	44 (100)	0 (0)	1 (2.3)	9 (17.0)	−	−	9 (100)	0 (0)	0 (0)	0 (0)	−	−	0 (0)	0 (0)	0 (0)
Italy-Lombardy	9 (15.5)	0 (0)	0 (0)	9 (100)	0 (0)	0 (0)	46 (79.3)	8 (17.4)	4 (8.7)	34 (73.9)	0 (0)	0 (0)	3 (5.2)	0 (0)	0 (0)	3 (100)	0 (0)	0 (0)
Italy-Tuscany	74 (53.6)	0 (0)	1 (1.4)	73 (98.6 )	0 (0)	1 (1.4)	52 (37.7)	6 (11.5)	2 (3.9)	44 (84.6)	2 (4.6)	2 (4.6)	12 (8.7)	8 (66.7)	0 (0)	4 (33.3)	0 (0)	0 (0)
Malta-MCAR^b^	11 (32.4)	−	1 (9.1)	10 (90.9)	0 (0)	0 (0)	16 (47.1)	−	5 (31.3)	11 (68.8)	0 (0)	0 (0)	7 (20.6)	−	1 (14.3)	6 (85.7)	0 (0)	0 (0)
Mexico-RYVEMC^b^	215 (40.0)	−	6 (2.8)	209 (97.2)	12 (5.7)	2 (1.0)	190 (35.3)	−	20 (10.5)	170 (89.5)	51 (30.0)	10 (5.9)	133 (24.7)	−	41 (30.8)	92 (69.2)	22 (23.9)	10 (10.9)
Netherlands-Northern	83 (36.9)	2 (2.4)	0 (0)	81 (97.6)	1 (1.2)	1 (1.2)	113 (50.2)	23 (20.4)	9 (8.0)	81 (71.7)	9 (11.1)	16 (19.8)	29 (12.9)	1 (3.5)	3 (10.3)	25 (86.2)	3 (12.0)	2 (8.0)
Slovak Republic	149 (56.4)	0 (0)	0 (0)	149 (100)	0 (0)	1 (0.7)	108 (40.9)	0 (0)	2 (1.9)	106 (98.1)	0 (0)	20 (18.9)	7 (2.7)	0 (0)	0 (0)	7 (100)	0 (0)	2 (28.6)
South America	511 (31.2)	−	6 (1.2)	505 (98.8)	9 (1.8)	11 (2.2)	1,127 (68.8)	−	153 (13.6)	974 (86.4)	335 (34.4)	61 (6.3)	0 (0)	−	−	−	−	−
Spain-ECEMC^f^	255 (43.4)	−	1 (0.4)	254 (99.6)	2 (0.8)	0 (0)	274 (46.7)	−	29 (10.6)	245 (89.4)	45 (18.4)	7 (2.9)	58 (9.9)	−	3 (5.2)	55 (94.8)	8 (14.5)	2 (3.6)
Sweden	688 (51.7)	3 (0.4)	1 (0.2)	684 (99.4)	4 (0.6)	3 (0.4)	524 (39.4)	81 (15.5)	8 (1.5)	435 (83.0)	21 (4.8)	31 (7.1)	118 (8.9)	8 (6.8)	1 (0.9)	109 (92.4)	6 (5.5)	8 (7.3)
Ukraine-OMNI-Net	51 (51.5)	2 (3.9)	1 (2.0)	48 (94.1)	1 (2.1)	0 (0)	46 (46.5)	1 (2.2)	7 (15.2)	38 (82.6)	1 (2.8)	2 (5.6)	2 (2.0)	0 (0)	0 (0)	2 (100)	0 (0)	0 (0)
UK-Wales	87 (33.1)	1 (1.2)	1 (1.2)	85 (97.7)	0 (0)	0 (0)	153 (58.2)	65 (42.5)	6 (3.9)	82 (53.6)	5 (6.1)	5 (6.1)	23 (8.8)	11 (47.8)	0 (0)	12 (52.2)	1 (8.3)	2 (16.7)
USA-Utah	96 (39.5)	7 (7.3)	1 (1.0)	88 (91.7)	7 (8.0)	1 (1.1)	117 (48.2)	10 (8.6)	2 (1.7)	105 (89.7)	5 (4.8)	5 (4.8)	30 (12.4)	2 (6.7)	0 (0)	28 (93.3)	2 (7.1)	1 (3.6)
TOTAL	2,712 (41.5)	17 (1.2^g^)	34 (1.3)	2,661 (98.1)	38 (1.4^h^)	47 (1.8^h,i^)	3,287 (50.3)	282 (22.1^g^)	288 (8.8)	2,717 (82.7)	483 (17.8^h^)	284 (10.5^h,i^).	536 (8.2)	62 (21.5^g^)	56 (10.4)	418 (78.0)	43 ( 10.3^h^)	33 (7.9^h,i^)

*Note:* Czech Republic, Iran-TROCA, Mexico-Nuevo Leon, US A-Arkansas, US A-Atlanta, USA-Texas not included in this analysis due to lack of information on case status of ARM.

Abbreviations: ECEMC, Registry of the Spanish Collaborative Study of Congenital Malformations; ECLAMC, Latin American Collaborative Study of Congenital Malformations; ETOPFA, Elective Termination of Pregnancy for Fetal Anomalies; MCAR, Malta Congenital Anomalies Registry; OMNI-Net, Ukraine Birth Defects Prevention Program; REN AC, National Network of Congenital Anomalies of Argentina; RYVEMCE, Mexican Registry and Epidemiological Surveillance of External Congenital Malformations; SMC, Soroka Medical Center; TROCA, Tabriz Registry of Congenital Anomalies; USA, United States of America.

a A hyphen (–) means that the registry did not report follow-up data for that time period.

b ETOPFA not allowed.

c frequency and percentage refer to first week mortality.

d ETOPFA not registered.

e Data on liveborn children with ARM from one hospital.

f Spain included information on ETOPFA from 1995 to 2014. ETOPFA not routinely registered in all the participating hospitals.

g Excludes programs where ETOPFA is unavailable or does not report on ETOPFA.

h Excludes programs that have no data on mortality for selected age at death.

i Argentina is included under Day 2–6.

## Data Availability

The data that support the findings of this study are available on request from the corresponding author. The data are not publicly available due to privacy or ethical restrictions.
