# Supplementary material for: Prevalence and mortality among children with anorectal malformation: A multi-country analysis
Source: Birth Defects Res. Author manuscript; Available in PMC 2023 Feb 4. (PMC9898144; doi:10.1002/bdr2.2129)
Supplement: Supplement 1 [file NIHMS1852648-supplement-Supplement_1.docx]

**Table 2**. Description of follow-up method for live births from birth defects surveillance programs contributing to the anorectal atresia prevalence and mortality study, International Clearinghouse for Birth Defects Surveillance and Research (ICBDSR)

| **Country-Program** | **Follow-up until discharge from the maternity hospital** | **Follow-up by a clinician or program staff** | **Linkage with death certificates** | **Maximum follow-up period reported in the study** |
| --- | --- | --- | --- | --- |
| Argentina-RENAC | Yes | Only until discharge from maternity hospital | No | 2-6 days |
| Colombia- Bogotá | Yes | Yes | No | 1 day |
| Colombia-Cali | Yes | Yes | No | No mortality reported for live births |
| Czech Republic | No | No | Yes | ≥ 5 years |
| France-Paris | Yes | Yes | No | 7-27 days |
| Germany-Saxony Anhalt | Yes | Yes^1^ | No | ≥5 years |
| Iran-TROCA | Yes | Yes | No | 2-6 days |
| Israel-SMC | Yes | Only until discharge from maternity hospital | Yes, 2000 to 2014 | 28 days -11 months |
| Italy-Lombardy | No | No | Yes, 2003 to 2015 | ≥ 5 years |
| Italy-Tuscany | No | No | Yes, 1992 to 2015 | ≥ 5 years |
| Malta-MCAR | Yes | Yes | Yes | ≥ 5 years |
| Mexico-Nuevo Leon | Yes | No | No | 7-27 days |
| Mexico-RYVEMCE | Yes | Only until discharge from maternity hospital | No | 2-6 days |
| Netherlands-Northern | Yes | Yes | No | ≥ 5 years |
| Slovak Republic | Yes | Only until discharge from maternity hospital | No | 7-27 days |
| South America-ECLAMC | Yes | Yes | No | 28 days - 11 months |
| Spain-ECEMC | Yes^2^ | Only until discharge from maternity hospital | No | 2-6 days |
| Sweden | No | No | Yes, 1974 to April 2016 | ≥ 5 years |
| United Kingdom-Wales | Yes | No | Yes, to GP system, till 18 years | ≥ 5 years |
| Ukraine-OMNI-Net | Yes | Yes | No | 1-4 years |
| USA-Arkansas | Yes | Only until discharge from maternity hospital | Yes, 1993 to 2015 | ≥ 5 years |
| USA-Atlanta | Yes^3^ | Only until discharge from maternity hospital | Yes, 1979 to 2008 | ≥ 5 years |
| USA-Texas | Yes | Only until discharge from maternity hospital | Yes, 1996 to 2013 | ≥ 5 years |
| USA-Utah | Yes | Only until discharge from maternity hospital | Yes, until age 2 years | ≥ 5 years |

^1^ Until age of 18 years

^2^ The participating physicians in the program are especially trained on the ascertainment of birth defects

^3^ Only hospital admissions are followed until age six years

Abbreviations: ECEMC=Registry of the Spanish Collaborative Study of Congenital Malformations; ECLAMC=Latin American Collaborative Study of Congenital Malformations; ETOPFA=Elective Termination of Pregnancy for Fetal Anomalies; MCAR=Malta Congenital Anomalies Registry; OMNI-Net=Ukraine Birth Defects Prevention Program; RENAC=National Network of Congenital Anomalies of Argentina; RYVEMCE=Mexican Registry and Epidemiological Surveillance of External Congenital Malformations; SMC=Soroka Medical Center; TROCA=Tabriz Registry of Congenital Anomalies; USA=United States of America.
